# Association of functional variants of *PTPN22* and *tp53* in psoriatic arthritis: a case-control study

**DOI:** 10.1186/ar1880

**Published:** 2006-01-03

**Authors:** Christopher Butt, Lynette Peddle, Celia Greenwood, Sean Hamilton, Dafna Gladman, Proton Rahman

**Affiliations:** 1 Memorial University of Newfoundland, Hospital for Sick Children, Department of Public Health Sciences, University of Toronto, Toronto, Canada; 2 Genetics and Genomic Biology, Hospital for Sick Children, Department of Public Health Sciences, University of Toronto, Toronto, Canada; 3 University Health Network, Toronto Western Hospital, University of Toronto, Toronto, Canada

## Abstract

Recent studies have implicated *PTPN22 *and *tp53 *in susceptibility to several autoimmune diseases, including rheumatoid arthritis, suggesting that these genes are important in maintaining immune homeostasis. Because autoimmune diseases may share similar susceptibility loci, investigation of these genes in psoriatic arthritis (PsA) is of potential relevance. As a result we investigated known coding polymorphisms in *PTPN22 *and *tp53 *in a homogenous Caucasian PsA cohort from Newfoundland, Canada and an admixed Caucasian PsA cohort from Toronto, Canada. We observed a moderate association of the R620W variant of *PTPN22 *with PsA in the Toronto population only. Because of the conflicting findings reported regarding the association of *PTPN22 *with PsA, further studies in other PsA populations are warranted.

## Introduction

Recently, two novel genes have attracted attention in the investigation of autoimmune disease. The *PTPN22 *gene encodes a functional protein tyrosine phosphatase known as lymphoid phosphatase, which acts as a regulator of the negative regulatory kinase cytoplasmic tyrosine kinase in T cells, and may play a role in suppressing T cell activation [1]. A functional single nucleotide polymorphism (SNP) at nucleotide position 1858, causing an Arg→Trp substitution (R620W) that disrupts the binding site for cytoplasmic tyrosine kinase, was recently found to be associated with type 1 (insulin dependent) diabetes [2]. Subsequently, associations were also found with other autoimmune diseases, including rheumatoid arthritis (RA) [3] and systemic lupus erythematosus [4] in Caucasian populations. A large study in psoriasis involving 1,146 affected individuals [5] and a smaller study in psoriasis in 265 families with multiple autoimmune diseases [6], with only 63 psoriatics, revealed no association of the R620W variant of *PTPN22 *with psoriasis.

The p53 protein has long been known to be related to the regulation of cell growth and prevention of carcinogenesis. It was recently shown that *tp53 *is consistently underexpressed in several autoimmune diseases, including RA, systemic lupus erythematosus, multiple sclerosis and type 1 diabetes [7]. Furthermore, the cellular damage response pathways that are dependent on p53 are defective in patients with RA [8]. A functional variant of p53 (Pro72Arg) has been shown to induce apoptosis markedly better than the wild-type variant, and has been associated juvenile chronic arthritis [9] but not with adult-onset RA [10].

Although psoriasis and psoriatic arthritis (PsA) are interrelated disorders, PsA is a distinct entity with its own epidemiological, clinical and genetic features. Furthermore, PsA exhibits much greater heritability among first-degree relatives (λ_1 _48) than does psoriasis (λ_1 _5–10) [11]. Therefore, we set out to examine the association between these two high priority candidate genes in two well characterized Caucasian PsA cohorts.

## Materials and methods

This study was approved by the local ethics committee of the Memorial University of Newfoundland and University of Toronto. Informed consent was obtained from all patients. All PsA probands were Caucasians. Information was collected systematically and included age at onset of psoriasis and PsA, and disease pattern. The control individuals (controls) were of similar ethnicity to the patients (cases). Controls for the Newfoundland population were volunteers from Newfoundland who participated in our study as a result of a local campaign seeking population-based controls for genetic studies. The Toronto controls were ascertained from the local HLA laboratory DNA bank, which includes healthy volunteers and organ donors.

Whole blood samples were obtained from PsA probands and control individuals. DNA was extracted using the Promega Wizard Genomic DNA purification Kit (Promega Corporation, Madison, WI, USA). Detection of SNPs was performed by analyzing primer extension products generated from previously amplified genomic DNA using a Sequenom chip-based MALDI-TOF mass spectrometry platform (Sequenom Inc., San Diego, CA, USA). In brief, polymerase chain reaction and extension reactions were designed using MassARRAY design software (Sequenom Inc.) and were carried out using 2.5 ng template DNA. Unincorporated nucleotides in the polymerase chain reaction product were deactivated using shrimp alkaline phosphatase. The amplification of the SNP site was carried out using the MassExtend primer and involved the use of specific d/ddNTP termination mixes, which were also determined using MassARRAY assay design software. The primer extension products were then cleaned and spotted onto a SpectroChip (Sequenom Inc.). The chips were scanned using a MT Analyzer (Bruker Daltonics Inc., Billerica, MA, USA) and the resulting spectra were analyzed and genotypes were determined using the Sequenom SpectroTYPER-RT software. We genotyped PsA probands and control individuals for the following polymorphisms: *PPTN22 *(rs2476601, R620W) and *tp53 *(rs1042522, Pro72Arg).

To determine differences in allele and genotyping frequencies, 2 × 2 contingency tables were used. Power calculations were performed by simulating cases and controls assuming a multiplicative model for disease risk, and varying the genetic risk associated with each copy of the high-risk variant. Observed allele frequencies among controls were used to generate genotypes, together with an assumed baseline risk for PsA of between 0.005 and 0.01. For each candidate gene, 100 simulated data sets were created, the trend test was performed, and we counted the number of simulations in which the *P *value was less than 0.05.

## Results

A total of 238 Newfoundland PsA patients and 149 healthy Newfoundland control individuals were studied. With respect to the Newfoundland PsA patients, 53% were male and their mean age at onset of the study was 49.7 years. The mean age at onset of psoriasis was 29.3 years (standard deviation 14.2 years) and the mean age at onset of PsA was 38.1 years (standard deviation 11.0 years). Of the PsA patients, 60% had polyarticular disease, 32% had oligoarticular disease and 7% had an isolated spondyloarthropathy. For the Toronto population, 207 PsA patients and 203 control individuals from the Toronto population were genotyped. With respect to Toronto PsA patients, 61% were male and their mean age at the start of the study was 39.6 years (standard deviation 11.3 years). The mean age at onset of psoriasis was 26.8 years (standard deviation 12.1 years) and the mean age at onset of PsA was 33.0 years (standard deviation 10.8 years). Forty-four per cent of the PsA patients had polyarticular disease, 40% had oligoarticular disease and 2.9% had isolated spondyloarthritis.

Of the 238 PsA patients genotyped for the R620W variant of *PTPN22 *in the Newfoundland cohort, the C/C, C/T and T/T genotypes for cases were 191, 44 and 3, respectively. For the 149 controls, the C/C, C/T and T/T genotypes were 121, 25 and 3, respectively. There was no difference in the minor allele (T) frequency between cases (10.5%) and controls (10.4%) for this *PTPN22 *variant (*P *= 0.96).

A total of 207 PsA patients and 199 control individuals were genotyped in the Toronto population for the R620W variant of *PTPN22*. For the PsA patients the G/G, G/A and A/A genotypes were 153, 43 and 7, respectively, whereas for the Toronto control individuals they were shown to be 167, 30 and 2. The minor allele (T) exhibited a frequency of 13.8% in PsA patients versus 8.5% in control individuals. This was statistically significant when tested for the minor T allele (*P *= 0.018) and for a trend in the genotypes (*P *= 0.024). Rheumatoid factor positivity was identified in 9% and 10% of the Newfoundland and Toronto cohorts, respectively. No association was associated with rheumatoid factor positivity and the minor (T) allele for the *PTPN22 *variant in either population.

With respect to Pro72Arg variant of *tp53*, 207 PsA patients were genotyped for the *tp53 *variant, and the G/G, G/C and C/C genotypes for PsA patients were 119, 87 and 11, respectively. For the 148 control individuals the G/G, G/C and C/C genotypes were 78, 60 and 10, respectively. There was no difference in the minor allele (C) frequency for this *tp53 *variant for cases (25.1%) and controls (27.0%; *P *= 0.56). The Toronto cohorts were genotyped for the *tp53 *variant in 205 PsA patients, resulting in 116 G/G, 76 G/C, and 13 C/C genotypes. With respect to the Toronto control individuals, 111 G/G, 76 G/C and 16 C/C genotypes were noted. The minor allele frequency of the Pro72Arg *tp53 *gene variant in cases and controls was 24.9% versus 26.6% (*P *= 0.57).

All control genotypes were in Hardy-Weinberg equilibrium. Using the minor allele frequency of 0.10, observed among the controls for the *PTPN22 *gene, the study had more than 85% power to detect a genotype relative risk of 2.0 or greater at *PTPN22*, and a power near 0.67 to detect a genotype relative risk of 1.75. At the *tp53 *gene, in which the minor allele frequency among controls was higher (0.27), the power estimates were near 0.75 for a genotype relative risk of 1.5, and 0.95 for genotype relative risks of 1.75.

## Discussion

This is the first study to assess the association of the high priority candidate genes *PTPN22 *and *tp53 *specifically in PsA. With respect to the R620W variant of *PTPN22 *in the Newfoundland population, our results are consistent with the reported studies in psoriasis [5,6]. However, a modest association was noted between this *PPTN22 *variant and PsA in the Toronto cohort. Because the Toronto cohort is the first population to report a significant association between *PTPN22 *and PsA and contradicts previous larger studies in psoriasis [5,6], this result should be interpreted with caution until it is independently validated in another PsA population. It is conceivable that a true association exists and that this association is disease (PsA) and population (Toronto) specific. It is worthwhile noting that the lymphoid-specific phosphatase encoded by *PTPN22 *is among the most powerful inhibitors of T cell activation, and so there is a potential rationale for this association. Alternatively, a false-positive association may have occurred in the Toronto PsA cohort because of population stratification. Because the reported RA associations with *PTPN22 *are almost exclusively with seropositive RA [3,6,9,10], we stratified our population based on seropositivity for rheumatoid factor, and found no association with *PTPN22*.

Over-expression and functional mutations of p53 have been noted in synovial tissues of RA [12] and in cutaneous lesions of psoriasis [13]. Because PsA shares pathogenic mechanisms with RA and psoriasis, Salvador and coworkers [14] examined p53 protein expression in synovial tissue of patients with RA and PsA. They reported differential p53 expression in the synovium of patients with RA as compared with PsA synovium. PsA patients had much less protein expression. This suggests a different pathogenic mechanism in PsA as compared with RA, and our study lends further support to this contention because no association with *tp53 *was noted in either of our PsA cohorts.

## Conclusion

In this study we investigated the association of *PTPN22 *in two independent PsA cohorts and obtained conflicting results. A moderate association was noted in a well characterized, admixed PsA cohort from Toronto, but this was not validated in a homogenous Caucasian cohort from Newfoundland. Therefore, further studies in additional PsA populations are warranted to determine more definitively the role of *PPTN22 *in PsA. No associations were observed with *tp53 *in either population.

## Abbreviations

PCR = polymerase chain reaction; RA = rheumatoid arthritis; SNP = single nucleotide polymorphism.

## Competing interests

The authors declare that they have no competing interests.

## Authors' contributions

CB carried out molecular genetic studies, participated in study design, and drafted the initial manuscript. LP assisted in genotyping some of the control individuals. SH aided in recruitment and clinical phenotyping of PsA patients. PR and DG conceived the study, participated in its design and coordination, and helped to draft the manuscript. CG performed the statistical analysis and revised the manuscript. All authors read and approved the final manuscript.
